# Effect of Finishing Diet and Lairage Time on Steers Welfare in Uruguay

**DOI:** 10.3390/ani11051329

**Published:** 2021-05-07

**Authors:** Marcia del Campo Gigena, Juan Manuel Soares de Lima, Gustavo Brito, Xavier Manteca, Pilar Hernández, Fabio Montossi

**Affiliations:** 1 INIA Tacuarembó, Ruta 5 km 386, C.P.45000 Tacuarembó, Uruguay; jsoaresdelima@inia.org.uy (J.M.S.d.L.); gbrito@inia.org.uy (G.B.); fmontossi@inia.org.uy (F.M.); 2 Universidad Autónoma de Barcelona, 08193 Bellaterra, Spain; xavier.manteca@uab.es; 3 Universitat Politècnica de València, Camino de Vera s/n, 46022 Valencia, Spain; phernan@dca.upv.es

**Keywords:** stress response, transport in cattle, lairage time, temperament

## Abstract

**Simple Summary:**

The transport and general handling of slaughter animals are associated with a series of events that cause stressful and unfavorable conditions that can compromise animal welfare. All these stressful events start at the farm and end with the death of the animal. In this experiment, we evaluated the effect of two finishing strategies and two contrasting lairage times through the combination of several indicators regarding productivity, physiology, behavior and postmortem variables. Individual temperaments and their impact on welfare and carcass quality were also considered. Animal welfare was not compromised in any diet during the finishing period. Individual temperament had a positive impact on the productivity and on all physiological indicators at different preslaughter stages. For that reason, we consider that it should be given paramount importance when talking about animal welfare. According to our results, with pasture-based animals, without fasting on the farm and after a short time of transportation (3.5 h), a longer preslaughter resting time (15 vs. 3 h) is desirable from the animal welfare perspective. Furthermore, our results suggest that this longer resting period, would also be more convenient from the carcass quality perspective.

**Abstract:**

The objective of this experiment was to evaluate the effect of two different pasture-based finishing strategies and lairage time on steers welfare in Uruguayan conditions. Sixty Hereford (H) and Braford (B) steers were assigned to two different diets for finishing purposes: (D1) native pasture plus corn grain (1% of live weight) (H *n* = 15, B *n* = 15) and (D2) high-quality pasture (H *n* = 15, B *n* = 15). The average daily gain was registered every 14 days, and temperaments were individually assessed one week before slaughter by three individual tests: crush score, flight time and exit speed, building a multicriterial temperament index (TIndex). Animals were slaughtered the same day in two groups (50% from D1 and 50% from D2 in each group) after traveling for 3.5 h and staying 15 (long lairage) and 3 h (short lairage) in the lairage pens, respectively. The behaviors were observed during lairage, and physiological indicators were used to assess stress at the farm after transport, after lairage and at slaughter. Bruises incidence and final pH were registered at the abattoir as a means of assessing the overall animal welfare. Calmer animals had higher average daily gains with no differences either between diets or between breeds. Calmer animals also had a lower stress response during all preslaughter stages, regardless of the time in lairage. Transport did not imply psychological stress (cortisol) for any slaughter group, but physical stress was evident after transport in both groups through NEFA and CPK increases. Bruise incidences did not differ between lairage groups. The short lairage group did not have enough time to cope with the environment before slaughter, with the consequent deleterious effects on the carcass pH. Animals from the long lairage group had a higher metabolic response shown through NEFA values, but they had enough time to rest and recover overnight, reaching final pH values lower than 5.8, considered the upper limit of the normal range. According to this experiment, with pasture-based animals without fasting on the farm and after 3.5 h of transportation, a resting period of 15 h in lairage should be better than a 3-h one.

## 1. Introduction

The transport and handling of slaughter animals are associated with a series of events that cause stressful and unfavorable conditions that can compromise animal welfare, increase the chance of spreading disease [1,2] and reduce the meat quality [3,4]. All these potentially stressful events start at the farm and end with the death of the animal. They involve physical stress like food deprivation; fatigue due to transport to the abattoir; collision with equipment and psychological stress because of gathering and mixing, lairage and repeated handling, unfamiliarity to the environment and social disturbance because of the disruption of the rearing group [5]. Stress, whether physical or psychological in origin, induces behavioral and physiological changes [5] that can have a significant impact also on the quality of meat via their effects on muscle energy metabolism [6].

The significant relationship between preslaughter stress and meat quality has been widely documented [5,7,8,9,10,11,12,13]. Regarding the effects of lairage time on animal welfare and meat quality, controversial experimental results have been reported, depending on the production systems and the general context of the meat production chain. Several authors sustain that the time in lairage brings about several positive benefits and potentially allows cattle to replenish muscle glycogen concentrations, reduce the dehydration of body tissues and carcass weight loss and to rest and recover from the effects of transport [14,15,16,17,18,19]. Other authors have reported that the lairage environment itself may inhibit the ability of cattle to rest or recover from the effects of feed and water restriction [20,21,22,23]. These varying results should be expected, given the multifactorial characters of these traits, leading the different study designs (preloading fasting at the farm, transport distance and transport time and lairage conditions) to produce different outcomes [14].

In this context, strict regulations and directives have been issued to promote animal welfare during preslaughter stages and some international bodies, probably, not considering either the mentioned different realities or differences among species (mainly ruminant vs. nonruminants), which recommend that all livestock animals should be slaughtered immediately after their arrival at the abattoir [24,25]. Therefore, in many European countries, it is common to slaughter animals on the day of arrival, whereas, in South America, due to the climatic, geographic and sociocultural conditions, among other factors, is common that animals are slaughtered the day after arrival. In turn, within South America, there are different country sizes, vast differences between countries in livestock transport durations and in the average distances between farms and abattoirs [26,27] and in preslaughter lairage time regulations. In Uruguay, where meat production is mainly based on grasslands, because of national meat safety regulations, animals are more typically slaughtered the day after arrival, reaching 12 h in lairage as the mean [28] and after having traveled relatively short distances from the farms to the packing plants (250 km in average) [29].

In this context and looking for a proper lairage duration in Uruguayan conditions, the objective of this experiment was to evaluate the effects of different pasture-based finishing strategies and two contrasting lairage times on steers welfare. The relationships between temperament and variables related to animal welfare were also assessed in the present study.

## 2. Materials and Methods

This study was run by the National Institute of Agricultural Research at INIA Tacuarembó Research Station, Tacuarembó, Uruguay (Latitude South 32°02′12.4″; Longitude West 57°09′15.2″) over a period of 5 months (through the end of summer, autumn and the beginning of winter). Sixty Hereford and Braford steers 2.5 years old were assigned to the following diets with finishing purposes according to live weight and breed: (D1) rangeland plus corn grain with the grain supplied at 1% of live weight (LW) (Hereford *n* = 15, Braford *n* = 15) and (D2) high quality pasture composed mainly of lotus (*Lotus corniculatus*) with a small proportion of white clover (*Trifolium repens*) (Hereford *n* = 15, Braford *n* = 15). In D1, *Paspalum notatum*, *Botriochloa laguroides*, *Stipa setigera* and *Paspalum dilatatum* made up 2/3 of the paddock total forage production. The area for each finishing strategy (35 hectares, 1.16 hectares/animal) was divided into two plots by electric fencing and animals alternated plots every 14 days. The system was planned in order to avoid overgrazing.

### 2.1. Field Determinations

#### 2.1.1. Productivity

Animals were weighed early in the morning without previous fasting every 14 days. For D1, amounts of corn grain were adjusted at this time according to LW. The supplement was provided once a day early in the morning (6 a.m.). Animals from both finishing strategies had ad libitum access to water.

#### 2.1.2. Temperament

Hair whorl position (HWP) was recorded on the first day of the experiment, looking for a correlation with temperament. If the center of the hair whorl was above the top of the eyes, the animal was categorized as “excitable”, “medium” if the center was located at eye level and “calm” if the center was located below the bottom of the eyes [30].

Individual temperament was assessed one week before slaughter by 3 individual tests: Crush score (CS), Flight time (FT) and Exit speed (ES): (a) (CS)—the animal behavior is scored while it is in a chute, using a 1 (calm)–5 (combative) scale, adapted from Hearnshaw and Morris [31]. The categories took into account the general state of the animal, including movements of limbs, head and tail, as well as behavioral signs of stress, attributing one of the following scores: (1) animal does not offer resistance, remaining with tail, head and relaxed ears; (2) animal has little limb movement, keeps head up and ears erect; (3) animal has frequent but not vigorous movements of limbs, head, ears and tail; (4) animal offers great resistance, with sudden movements of head and tail, can jump and fall, with audible breathing; (5) paralyzed animal, with muscle tremor (freezing). The measurement was performed after the animal entered the chute. Only the rear (entrance) and front (exit) gates remained closed for the test, without the use of any of the containment structures (side walls, fisheries and coasters). The records were taken 4 s after closing the gates; (b) (FT)—the amount of time (in seconds) it takes an animal to cover a known distance (5 m) immediately after leaving a confinement situation was recorded. A manual stopwatch was used, and registration started when the chute gate was opened, and the animal had the chance to exit. Animals with shorter flight times were considered more excitable; and (c) (ES)—data were obtained through a nominal scale scoring cattle exit gait: 1 (walk), 2 (trot) and 3 (canter). Animals that canter were considered more excitable. A multicriterial temperament index (TIndex) was built from (FT), (CS) and (ES), following Saaty [32]. For that, a matrix was established with the relative importance of the (FT), (ES) and (CS) characteristics to each other, according to our criteria. This matrix was normalized. A standardized ranking of the animals was generated for each of the variables, on a scale from 1 to 100. Then, the index was constructed according to the following equation:

$$\mathrm{TIndex}\;=\;\overset{j}{\underset{1}{\sum }}W_{j}d_{i}$$

where “W” is the weight of each of the variables according to the researcher’s criteria applying Analytic Hierarchy Process-AHP [32], and “d” is the value of each normalized record. Considering that (FT) is an objective test, it was assigned a relatively higher ranking in the index, meaning that the higher the TIndex, the calmer the animal.

#### 2.1.3. Health Status

Pathological event or trauma and the corresponding medical treatments were daily observed and registered throughout the entire experimental period.

### 2.2. Transport and Slaughter Plant

All animals were slaughtered the same day in a commercial abattoir licensed to export meat, following standard animal welfare procedures. Each slaughter group was composed of 50% of animals from D1 and 50% from D2, remaining in pens for 3 (short lairage) and 15 h (long lairage) preslaughter, respectively. Animals from both slaughter groups remained grazing until loading (without fasting on the farm) and transported for 3.5 h in a commercial truck with two compartments, allowing 420 kg/m^2^ (1–1.2 m^2^/head) according to the abattoir protocol (based on international recommendations). Steers from different diets within each slaughter group were not mixed either in the truck or at the abattoir. The same truck and driver were used for both journeys. Distance from the farm to the slaughterhouse was 140 km, and the average driving time was 3 and a half h, with 3 stops of 3 to 4 min for animal monitoring. No problems were registered during loading and unloading, being fluid in both groups. After arriving at the abattoir, animals from each diet (*n* = 15) within each slaughter group were taken to a 37.5-m^2^ pen with 2 divisions (8 and 7 animals per division). The space allowance in lairage pens was 420 kg/2.5 m^2^, according to the protocol mentioned above. Animals from the long lairage group waited from 3 p.m. of day 1 to 6 a.m. of day 2 (slaughter day), and those animals from the short lairage group waited in lairage during the morning of day 2 (from 10 a.m. to 1 p.m.), being the first and the last group sacrificed that same day in the abattoir, respectively.

#### 2.2.1. Physiological Indicators

Three blood samples were taken four times from all animals, looking for basal values in welfare indicators and their respective changes, according to the following periods:

- Time A: before leaving the farm (basal values)
- Time B: immediately after arriving at the slaughterhouse (transport effect)
- Time C: after lairage (lairage effect)
- Time D: during bleeding immediately post slaughter (effect of the last handling procedures)

For bleeding, animals were conducted to a portable chute strategically located near the pens of both slaughter groups. One of the three samples was collected into anticoagulant (Becton, Dickinson and company, Franklin Lakes, New Jersey, USA) cooled and immediately sent for hematocrit determination. The other 2 samples were kept cool until they arrived at the laboratory. Serum was extracted following centrifugation at 3000 rpm for 15 min. The serum fractions were frozen and immediately sent for analysis:

Sample 1. Hematocrit. It was determined by the micro hematocrit technique at the University Veterinary Faculty in Uruguay. Results are expressed in percentages.

Sample 2. Cortisol and Creatine kinase (CPK). Serum samples were assayed in the Nuclear Techniques Laboratory at the University Veterinary Faculty in Uruguay.

Cortisol. Method: it was determined by a direct solid-phase radioimmunoassay (RIA) using DPC kits (Diagnostic Product Co., Los Angeles, CA, USA). All samples were determined in the same assay. The RIA had a sensitivity of 8.2 nmol/L (0.91 log nmol/L). The intra-assay coefficients of variation for low (36 nmol/L–1.56 log nmol/L), medium (224 nmol/L–2.35 log nmol/L) and high (427 nmol/L–2.63 log nmol/L) controls were 10%, 6.8% and 4.6%, respectively. Results are expressed in log nmol/L.

CPK. Method: CK NAC liquid UV. Liquid test for creatine kinase determination (EC 2.7.3.2.) activated by NAC and measured by spectrophotometry at 340 nm. Results are expressed in U/L.

Sample 3. Non-esterified fatty acids (NEFA) and ß-hidroxibutirate (βHB). Serum samples were assayed at the Rubino Laboratory (Ministry of Agricultural affairs) in Uruguay.

NEFA. Method: ACS-ACOD (acil-CoA sintetasa-acil-CoA oxidasa). WAKO laboratory kits (WAKO Chemicals, Richmond, VA, USA) were used (references 999-34691, 995-34791, 991-34891 and 993-35191)—lots TK 365, TK 366, TK 367 and TK 368. This method was adapted for use in a VITALAB Selectra 2 Autoanalyzer (Wiener Lab Group, Buenos Aires, Argentina). Results are expressed in nmol/L.

βHB. Method: D-3-hidroxybutyrate oxidation into acetoacetate through the 3-hidroxibutirate dehydrogenase enzyme. As a consequence, NAD+ from the reactive is reduced to NADH, and the absorbance changed to 340 nm. RANDOX laboratory kits were used (reference RB 1008)—094293 in a VITALAB Selectra 2 autoanalyzer. Results are expressed in nmol/L.

#### 2.2.2. Behavior in Lairage Pen

Cattle behavior was evaluated by 8 trained observers working in pairs, who rotated between divisions each hour to minimize the observer effect. Direct observation was performed within each pen division (experimental unit) combining the instantaneous scan sampling and the behavior sampling techniques [33]. Due to operative restrictions, animals were observed for 1.5 h in the short lairage and 7.5 h in the long lairage group. At each scan, the following behaviors (body postures and activities) were recorded: walking (without rumination-wr), lying (wr), standing (wr), ruminating, drinking water, conflicts (bumps with the head and mounting), positive social behavior and self-grooming. Results from the scan are shown as a percentage of the total time spent on each behavior. Conflicts are considered very important from the welfare perspective, being relevant to record each occurrence. Due to their possible short duration, these events would tend to be missed by scan sampling [33]. Therefore, in this experiment, conflicts (bumps with the head and mounting) were also registered with the behavior sampling technique at each pen division, between 2 scan periods. Each consecutive sample interval took 7.5 min. Animals were individually identified with a number painted on both sides of the body.

#### 2.2.3. Carcass Traits Indicators at the Abattoir as a Means of Assessing Overall Animal Welfare

##### Bruising

Before carcasses were dressed, they were visually inspected, recording the number and severity of bruises at the individual level. Severity was scored as major or minor, depending on whether they involved tissue remotion (minor: subcutaneous or no tissue remotion; major: affecting muscle).

##### pH

Carcass pH was measured at 24 h post-mortem (pm) at the Longissimus dorsi (LD) between the 12 and 13th ribs, using a pHmeter (Orion 210A; Cole-Parmer, Vernon Hills, IL, USA) with a gel device.

### 2.3. Statistical Analysis

Exploratory analyses were performed for all variables using Statgraphics (Statgraphics Technology Inc., The Planes, VA, USA) and SAS packages (SAS Institute Inc., Cary, NC, USA).

Productivity. A general linear model (PROC GLM) [34] was used to determine the effects of diet, breed and TIndex on ADG. Initial and final liveweights were included in the model as covariates. Interactions were considered and, if not significant, were removed from the model. ADG means were compared by the least-squares method (PROC LSMEANS) [34].

Physiological data. Due to absence of normality, the cortisol and CPK values were normalized by taking a natural logarithm. The effects of diet, breed and TIndex on physiological indicators through time (4 consecutive times) were evaluated through analysis of variance using a mixed model with repeated measures considering the animal as a random effect inside each diet (PROC MIXED) [34]. Initial and final liveweight were included in the model as covariates. As explained above, bleed samples were obtained four consecutive times. To model the correlation between repeated measures for each animal, a general linear mixed model was used (PROC MIXED) [34]. For each physiological indicator, means were compared by the least-squares method (PROC LSMEANS) [34].

For ADG and physiological indicators, several covariance structures were tested (variance components (VC), first-order autoregressive structure (AR (1)) and compound symmetry (CS)), in order to fit the best model. Goodness of fit was defined by the lower Akaike’s Information Criteria (AIC) value [35]. After each model was adjusted, robustness was tested, excluding from data standardized, residual values higher than 2 and lower than −2. The model was considered robust when explanatory variables stayed in the model after data filtering and model rerunning.

A regression analyses was performed to evaluate TIndex, lairage duration and final liveweight effects on cortisol concentration during slaughter.

Behavioral data. Scan sampling technique: Binomial data from each activity was modeled assuming a binary distribution and a logit link function, using the pen division as the subject/experimental unit. A general linear mixed model was used to study the effect of lairage time, diet, breed and temperament, on the frequency of each behavior (PROC GLIMMIX) [34]. Conflicts data from the Behavior technique was modeled, and a log-link function was set, assuming a Gamma distribution. A general linear mixed model was used to study the effect of lairage time, diet, breed and TIndex on conflicts in the first hour in lairage (PROC GLIMMIX) [34]. Hypothesis tests (binomial proportion) were performed to analyze the differences in conflict frequency (number of events per hour) between consecutive and nonconsecutive hours in lairage.

Carcass quality. Bruising frequency was compared by the χ^2^ test (PROC FREQ) [34], and the regression analysis was performed to study the effect of independent variables on the bruising frequency (PROC LOGISTIC) [34] and pH values (PROC REG) [34]. pH means were compared by the least-squares method (PROC LSMEANS) [34].

## 3. Results and Discussion

Neither breed nor HWP had an effect on the evaluated variables. In turn, none of them were associated with temperament. Therefore, breed and HWP discussion is omitted in this paper.

### 3.1. Field Determinations

#### 3.1.1. Productivity

ADG did not differ between diets (0.63 ± 0.02 in D1 and 0.64 ± 0.02 in D2). The crude protein content of Uruguayan rangeland pastures seems not to be restrictive for animal production [36] covering cattle and sheep maintenance requirements [37], but low ADG, especially in autumn, are usually due to the unbalanced chemical composition of native pasture, with low energy availability for the digestive process [38]. In our experiment, grazing was not restricted in any finishing strategy, crude protein contents were above critical values (9.22% and 22% in D1 and D2, respectively) and energy restrictions in D1 were compensated by the energetic supplementation, providing the animals with adequate daily gains.

#### 3.1.2. Temperament and ADG

Average TIndex was not different between diets (61.2 ± 5.6 in D1 and 54.1 ± 5.6 in D2) (*p* > 0.05). Calmer animals had higher ADG regardless of diet and breed (*p* < 0.05). These results are consistent with Voisinet et al. [39], who reported that calmer *Bos indicus*-cross and *Bos taurus* cattle had higher ADG than steers with excitable temperaments. Barnett et al. [40] and Hemsworth et al. [41] sustained that a fall in the rate of growth is the consequence of a series of acute or chronic responses to human presence and is probably more accentuated in temperamental animals. Regardless of temperament, gentler animals are known to be less susceptible to stress generated by management practices in which the human presence is involved [42], and their productivity is therefore less affected. In this study, all animals had been subjected to good animal husbandry practices prior to and during the test, probably contributed to the satisfactory ADG levels obtained.

#### 3.1.3. Health Status

Health was compromised in two animals from D2 during the experiment, not being related to feed problems in none of the cases. Immediate and effective control measures were applied, with no incidence on ADG of the involved animals. Good physical health is undoubtedly a necessary condition for animal welfare. However, health is more than the absence of disease, and understanding the relationship between health and welfare depends on drawing inferences about subjective feelings such as pain, discomfort and distress [43]. Based on productive and behavioral observations, it was considered that these events did not have a strong negative impact on welfare, and both animals remained in the experiment. No deaths were registered during the experimental period.

### 3.2. Transport and Slaughter Plant

#### 3.2.1. Physiological Indicators

##### CORTISOL

Time A—cortisol at the farm.

**Figure 1:**
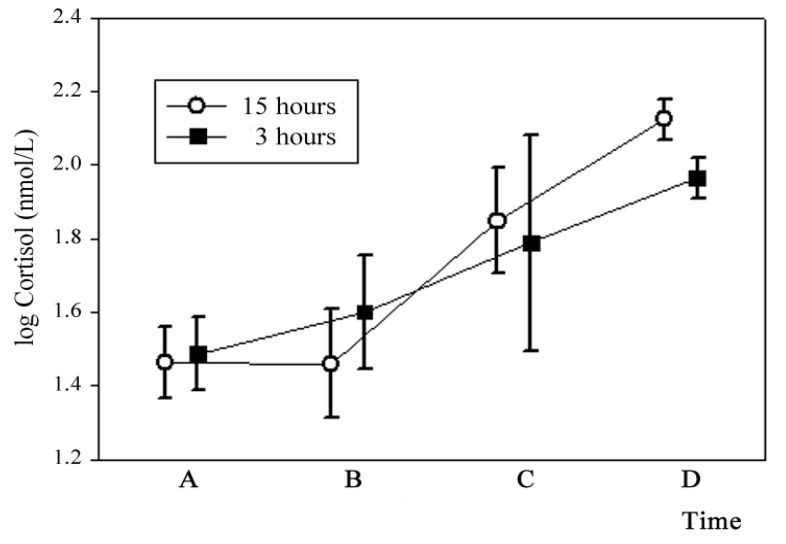
Serum cortisol (log) values at different times within each slaughter group. Lines represent media and confidence interval. Time A: farm basal value; Time B: after transport; Time C: after lairage; Time D: at slaughter.

Cortisol did not differ between diets at the farm (Figure 1).

Time B—Transport effect on cortisol.

Cortisol did not increase after transportation in any slaughter group. According to several authors, the major factors determining the welfare of cattle during road transport are: vehicle design, stocking density, trailer design and ventilation, driving and handling quality, transport duration, road and environmental conditions [12,44,45,46]. In our experiment, all these factors were standardized, and based on these data, it is assumed that the proper animal handling during all the preslaughter transportation process (including procedures at the farm, during transport and unloading) and the use of suitable equipment and facilities, contributed to our results. Similar results were reported by Ishiwata et al. [47], who did not find differences in plasma cortisol concentration before and after travelling, suggesting that transport had no severe effects on cattle. Fazio et al. [48] suggested that the effects of short-distance road transport on the increase in cortisol levels in cattle, probably depend on preliminary contact with staff during handling. Trunkfield and Broom [49] reported a sharp response in cortisol levels in calves during the first 2 h of transport, mainly due to the loading procedure. These authors suggested that cattle are stressed during the initial period of transportation (on short journeys) and that the degree of stress is greater after long-distance road transport. Villaroel et al. The authors of reference [50] also reported that cortisol was higher after 1 to 2 h of transportation compared to journeys that were less than 1 h or more than 2 h long. After this initial period on short journeys (less than 4 h), animals are thought to become accustomed to the new situation. In our experiment, animals from both slaughter groups showed a good habituation to transport (Figure 1, Time B). In short, on the basis of the comparative response of circulating levels of cortisol before and after transportation, our data do not agree with results that consider transport to be one of the most potent stressors for cattle [51]. That confirms that using best management practices for transportation will contribute to animal welfare, decreasing, and like in this case, avoiding the expected psychological stress response, even with animal coming from extensive conditions.

Cortisol concentrations did not differ between slaughter groups at Time B (Figure 1).

Time C—Lairage effect on cortisol.

Serum cortisol concentrations significantly increased respect to basal values after lairage in both lairage groups (*p* < 0.05; Figure 1, Time C). All animals were stressed, probably due to the inherent noises and movement of animals and people in the yards during routine handling at the abattoir. It is known that after a stressful event, hematological variables can return to basal levels within 30 min if animals are in their familiar environment [52]. In this study, probably due to the new environment, higher values of cortisol were registered even after 15 h in lairage. Cortisol concentrations did not differ between slaughter groups at Time C (Figure 1).

Time D—Preslaughter effect on cortisol.

Both slaughter groups also had a considerable preslaughter stress response, increasing to 1.95 and 2.11 log nmol/L in the short and the long lairage group, respectively (*p* < 0.05; Figure 1, Time D). This is consistent with results from Boissy and Le Neindre [53] and Lay et al. [54], who reported that cortisol levels in response to a stressor could increase up to 1.78–2.30 log nmol/L in cattle. Some authors believe that the increase in cortisol concentrations at Time D, during bleeding, are mainly a response to handling in the race when driving the steers to the stunning box [55] and that this stress depend on length and design of the chute and the quality of the human–animal relationship [11]. It is worth noting that they could also represent the cumulative effects of all stages of the preslaughter handling. Moreover, due to food safety requirements, cattle in Uruguay are washed on their way to the stunning box to remove hide or fleece contaminants, such as excreta and dirt. The process of handling and washing the animals would have elicited a stress response, which could partially explain the cortisol rise in both slaughter groups. Although the distance between washing and stunning is short, it could have been enough to raise HPA axis activity. In addition, the effect of the process of stunning itself cannot be disregarded. 

Cortisol concentrations did not differ between slaughter groups at Time D (Figure 1).

##### Temperament and Cortisol

**Figure 2:**
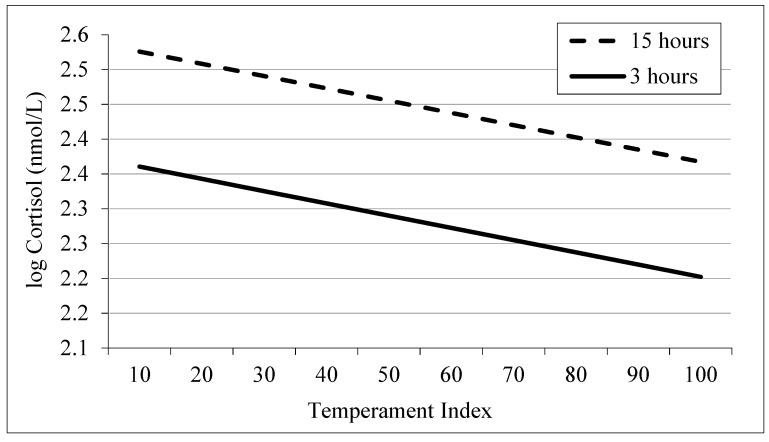
Average TIndex effect on (log) cortisol values at slaughter. Trendlines per slaughter group, estimated by regression analysis (R^2^ = 0.30).

Calmer animals showed lower cortisol concentrations in serum throughout the whole experiment (*p* < 0.05; Times A, B, C and D), regardless of diet or slaughter group. Figure 2 shows the effect of TIndex on log cortisol values at slaughter, where a relevant rise in cortisol was registered in both slaughter groups (*p* > 0.05). As it was previously mentioned, Time D could represent the cumulative effects of all stages of the preslaughter handling, implying that temperament and a proper handling are very important at this stage. Our results are consistent with those reported by Curley et al. [56], Café et al. [57] and Burdick et al. [58], who indicated that the functional characteristics of the HPA axis vary with animal temperament and sympathoadrenal-medullary responses can be more intense in excitable animals [57,59,60,61]. Stress may activate the pituitary-adrenocortical system [62], and these hormonal changes may affect cellular metabolic processes [63].

Our results provide support to the recognized influence of temperament in modulating the adrenal response of cattle to different stressful situations.

Cortisol concentrations did not differ between slaughter groups at Time D.

##### CPK

Time A—CPK did not differ between diets at the farm.

Time B—Transport effect on CPK.

Transportation had a significant effect on CPK (*p* < 0.05; Table 1; Time B). Absolute values in U/L increased 2 times at Time D, with respect to basal values in both slaughter groups, being consistent to several other studies [18,50,64,65,66].

CPK is a muscle-specific enzyme whose activity in the blood is useful for indicating leakage from the muscle as a result of trauma, physical exercise and stress and/or other muscle damage in animal production [67,68,69]. Transportation is a physical demanding factor, because animals have to maintain balance and the contact between animals produces fatigue and bruising, affecting the permeability of the muscle membranes and the liberation of the enzymes into the blood [11,68]. Even if driving is smooth, animals need to make a considerable physical effort during transportation to keep their balance (stability) and posture. Vibration and motion might also have caused stress. The increased activity of the enzyme in this experiment could represent possible trauma during loading, transport and unloading, or it could have increased as a result of behavioral interactions between steers [67,70].Therefore, in our experiment, traveling could have been an accumulation of the nonspecific stress response and the physical effort.

CPK concentrations did not differ between slaughter groups at Time B.

Time C—Lairage effect on CPK.

After lairage, CPK values did not increase in any slaughter group (Table 1, Time C). Similar results were found by Tadich et al. [55] who found higher CPK activity after transport (with 0, 3 and 16 h) but did not find an additional increase during lairage (in different combinations of transport: 0, 3, 16 h and lairage duration: 0, 3, 12, 16 and 24 h). Even considering that an important frequency of conflicts (bumps with the head and mounting) was registered during the first hour in lairage in both slaughter groups (see behavioral analysis), it was apparently not enough to increase serum CPK concentrations.

CPK concentrations did not differ between slaughter groups at Time C.

Time D—Preslaughter effect on CPK.

Preslaughter handling procedures had a significant effect on CPK (*p* < 0.05; Table 1; Time D). Absolute values in U/L increased four times at Time D, with respect to basal values in both slaughter groups. The higher presence of CPK implies constant muscle movement, both voluntary and those that are controlled by the autonomic nervous system (heart and lungs). Elevated plasma CPK activity is also associated with strenuous or unaccustomed muscular exercise [71]. For this reason, we considered that the stunning process itself could have had a considerable effect on our results (tonic and clonic phases).

CPK concentrations did not differ between slaughter groups at Time D.

##### Temperament and CPK

Calmer animals had lower CPK values in serum throughout the whole experiment (*p* < 0.05; Times A, B, C and D). Animals with the most excitable temperament are most susceptible to stress generated by routine handling practices, such as loading and unloading, transport or the new environment in the abattoir [72] with the consequent effects on CPK.

##### NEFA

Time A—NEFA did not differ between diets at the farm.

Time B—Transport effect on NEFA.

NEFA concentrations significantly increased respect to basal values after transportation in both slaughter groups (*p* < 0.05; Table 2; Time B). Similar results were found by Warris et al. [4], who reported that transport of cattle for between 5 and 15 h was associated with increases in blood concentrations of free fatty acids. Changes in these blood metabolites are indicative of body energy reserves mobilization, a mechanism necessary to maintain homeostasis [73]. Fasting and stressful events are typically associated with increased energy demands, and this leads to depletion of energy stores—in particular, liver glycogens and body fat [74]. Free fatty acids may also increase in response to catecholamine release following acute stress [75]. Although, in this experiment, cortisol concentrations did not increase after transport, it is possible that sudden moments of extremely acute stress (like sudden truck movements or vibrations) provoked the activation of the autonomic nervous system with the consequent increase in NEFA, although it was not enough to activate the HPA axis. According to Mellor and Stafford [76], the relatively slow response time of the HPA axis may make it insensitive as a means of discriminating different level of stress within the first few minutes after a noxious stimulus. The physiological changes elicited by the sympathetic adrenomedullary system may be more accurate in assessing the early stages of distress response [77]. In our experiment, physical stress was evident after transport, according to CPK and NEFA concentrations, but the results showed that the situation did not involve the HPA axis activity. The activation of the HPA axis is mainly dependent on the emotional involvement of the animal; stressors do not necessarily activate the HPA system when the animal does not perceive the situation as stressful [78]. Therefore, is not possible to conclude from the results obtained, that animals were suffering during transport. The physiological changes registered in this stage indicate that the adaptive mechanisms were functioning.

NEFA concentrations did not differ between slaughter groups at Time B.

Time C—Lairage effect on NEFA

After lairage, NEFA concentrations increased in the long lairage group (*p* < 0.05; Table 2; Time C), and NEFA values were higher than those of the short lairage group (*p* < 0.05), suggesting that food deprivation was not long enough to cause a lasting rise in NEFA in the short lairage. These results indicate a greater energy demand to restore homeostasis because of the longer food deprivation [79]. These differences could therefore be explained as a result of fat reserves being mobilized to supply energy requirements, probably with the additional effect of psychological stress due to the new environment, as shown in Figure 1. However, as has been mentioned, HPA axis activity increased but did not differ between groups during lairage.

Time D—Preslaughter effect on NEFA.

At slaughter, NEFA concentrations did not increase in any slaughter group (Table 2, Time D), but animals from the long lairage had greater NEFA concentration values at slaughter than the short lairage group (*p* < 0.05). Undoubtedly, the long lairage group presented higher energy demands. However, as has been mentioned, the HPA axis activity did not differ between slaughter groups at Time D, not being possible to infer more suffering in this group. Results from this experiment are consistent with those from Jarvis et al. (1996), who reported higher concentrations of NEFA during bleeding in animals that spent more than 16 h in the abattoir (overnight) when compared to animals that spent 5 h in lairage pens previous to slaughter (0.28 and 0.33 mmol/L, respectively). Cockram and Corley [80] also found that cattle held overnight in lairage had significantly greater plasma-free fatty acid concentrations than those slaughtered on the day of arrival.

##### Temperament and NEFA

Calmer animals had lower NEFA values throughout the whole experiment (*p* < 0.05; Time A, B, C and D). Results from all physiological indicators show that the magnitude and quality of the stress response will be greatly affected by individual differences [81] and that the stress response mechanisms are much more active in excitable animals than in their calmer counterparts.

Considering that temperament has been validated as a consistent trait that can be easily assessed on a farm [82] by direct observation [83] and due to its positive effect on all physiological indicators at different preslaughter stages, it should be given paramount importance when talking about animal welfare. 

##### β-HIDROXIBUTIRATE (βHB)

βHB did not differ between slaughter groups at any Time (A, B, C and D). Ketonic bodies, like βHB, are excellent fuel for tissue respiration—in particular, when glucose levels are limited (fasting). However, under these circumstances, these tissues can easily use NEFA energy sources. In the present study, probably fasting was not long enough to cause a strong and clear βHB stress response and to determine differences between slaughter groups.

#### 3.2.2. Behavior in Lairage Pen

**Figure 3:**
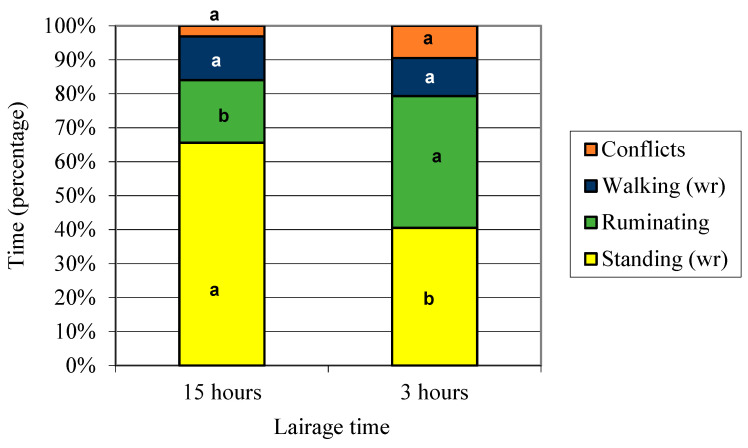
Percentage of the total time destined/allotted to each observed behavior by slaughter group. Note: The same activity with different letter (between bars) differs with *p* < 0.05.

According to the Scan sampling technique, animals did not drink water during lairage at the abattoir unless they had the opportunity to rehydrate after arrival [84]. It is possible that this behavior was suppressed as a result of unfamiliarity with the new environment, being consistent to several authors who reported that not all animals will drink water [8,85,86], as the priority is to settle down and explore the pen rather than drinking [22,55,87]. However, in the present experiment, hematocrit values at slaughter showed that animals were not dehydrated. If cattle are fully hydrated and fed before transport, it is likely that food deprivation rather than water will be the greater stressor over the initial 24 h, since this is more likely to disrupt rumen function [88]. In this study, animals did not perform any positive social behavior, self-grooming or lie down during the scan. Steers from both slaughter groups spent around 80% of total time in lairage, standing (wr) and ruminating (Figure 3), and no differences were registered in the percentage of time for walking (wr) and conflicts between slaughter groups. The short lairage group had a higher frequency of rumination (Figure 3, *p* < 0.05). Animals are known to ruminate while resting [89], and time spent ruminating is a direct indicator of animal welfare [90]. However, our results could be better explained by the experimental schedule defined to reach the stipulated preslaughter waiting hours, as mentioned in Section 2.2. Although grazing behavior is affected by various environmental conditions [91], most grazing behavior studies show similarity in daily grazing patterns, with the major grazing period occurring early in the morning and another later in the afternoon, with intermittent grazing occurring throughout other periods of the day and night (baseline ethogram) [92]. In the present experiment, the short lairage group took advantage of the grazing peak of the afternoon on the preslaughter day and kept grazing until dawn (it was loaded at 6 a.m. of the slaughter day). The 15 h group was loaded at 11 a.m. of the preslaughter day, not being able to perform the afternoon grazing peak on that day. Despite the aforementioned differences in the rumination frequency between laughter groups, high rumination frequencies were recorded in the long lairage group, up to the last hour of observation (7.5 h). A similar experiment developed in Uruguay with steers fed on the pasture and comparing 3 vs. 12 h in lairage, registered high frequencies of rumination until the tenth hour in lairage [93]. Results from both experiments suggest that animals did not experience the hunger sensation during the evaluated corresponding periods.

In the present experiment, results from the GLIMMIX procedure showed that animals from D2 spent more time ruminating than supplemented steers (D1) (*p* < 0.05) in both slaughter groups. These results could mainly be explained by the fact that animals from D2 were strictly fed on pasture (without supplementation), probably implying a higher fiber consumption, a slower digesta passage and, therefore, a larger rumination period [14,94].

According to the behavior sampling technique, conflicts frequency (bumps with the head plus mounting/hour) did not differ between slaughter groups, but results from the GLIMMIX procedure showed that regardless of the slaughter group, supplemented steers (D1) were more aggressive than those from D2 (*p* < 0.05). These results are consistent with the higher rumination time registered in animals from D2, in both slaughter groups.

**Figure 4:**
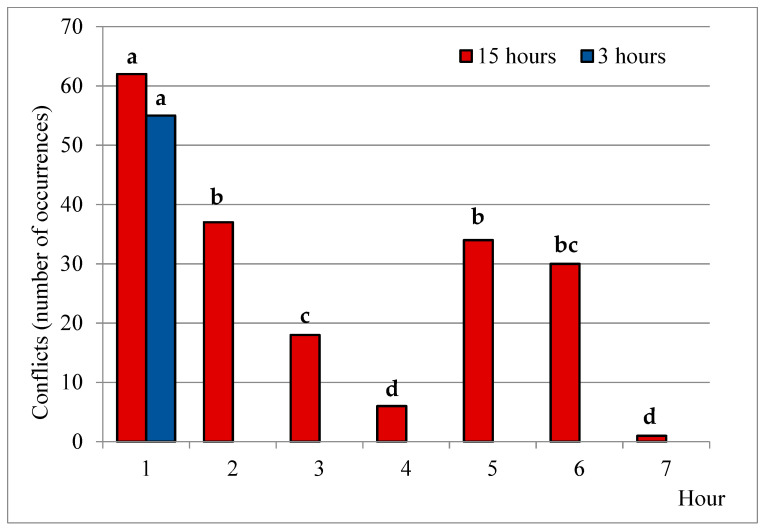
Number of conflicts during consecutive hours in lairage for each slaughter group. Bars with different letter differ *p* < 0.05.

When analyzing conflicts frequency during the first hour in lairage, no differences were found between slaughter groups (Figure 4). The frequency of this activity in consecutive hours (in the long lairage group) was therefore compared to conflicts frequency during the first hour. Results from each binomial proportion comparison showed that conflicts frequency in the first hour in pens was significantly higher than the second, third, fourth, fifth, sixth and seventh hours, respectively (Figure 4; *p* < 0.05). The first hour in pens was a critical adaptation stage for both groups, but animals that remained in pens became calmer afterwards. According to these results, we could have expected the same evolution in conflicts frequency in the short lairage group. The lowest conflicts frequency in the 15 h group (with respect to the first hour) was registered during the 4th and 7th hour (Figure 4, *p* < 0.05).

Both groups were situated in quiet environments far from the unloading facilities, but the long lairage group waited overnight, with greater opportunities to rest. Noise generated by the normal abattoir activity was noticeably higher during the morning and mid-day because of the slaughter procedures. This could have contributed to a higher excitability in the 3-h group, not having the opportunity to rest or to get used to the pens. However, considering that there were no differences between groups in conflicts frequency during the first hour of observation, we consider that lack of resting time was probably the most important reason for these results. Conflict may be beneficial in the long run but will still be unpleasant while it lasts [95], especially considering those animals that did not have enough time to cope with the new situation (3-h group).

##### Temperament and Behavior

TIndex did not have an effect neither on time budget nor on conflicts frequency during the first hour in lairage, suggesting that this first hour was a critical adaptation stage for all animals.

#### 3.2.3. Carcass Traits Indicators at the Abattoir as a Means of Assessing Overall Animal Welfare

##### Bruising

Incidence of bruising was not significantly affected by lairage time, with 14 bruises registered in the long lairage and 15 in the short lairage group, respectively. These results are not consistent with those of Mc. Nally and Warris [96], who reported higher bruise incidence in carcasses from cattle that remained for longer lairage periods. Results from the third Uruguayan Beef Quality Audit (2013–2015) show that, at the commercial level, 71% of the carcasses in Uruguay had at least one bruise [97], costing the Uruguayan cattle industry 13 million of dollars in lost carcass value, annually, being 37% of the total losses of the meat chain [97]. Bruises are a very good indicator of animal welfare, and when a bruise affects muscle tissue, the affected area is trimmed during postmortem processing, leading to economic losses due to decreased carcass value from reduced carcass yield and, depending on bruise location, potential devaluing of cuts [29,98]. In the present experiment, major bruises affecting the carcass and meat quality were only registered in the long lairage group (1 bruise in two animals) and both steers jumped through the chute while being bled. In spite of not having differences between slaughter groups, 50% of bruises incidence is very relevant, implying that Uruguay must identify causes and stages where bruises are provoked and strengthen corrective capacitation strategies for diminishing its incidence.

Temperament and Bruising.

TIndex was not related to bruise incidence. The good management practices followed during the whole experiment including the abattoir, could have contributed to these results. These results are not consistent to Barnett et al. [99] who reported that the vigorous avoidance response of cattle with poor temperament in confined areas during handling, transport and preslaughter increases the likelihood of falling and of collision with yard or stock crate structures and, also, with other cattle, increasing the chance of bruising.

##### pH

Carcasses from the short lairage group had higher values of final pH (5.83 ± 0.04 vs. 5.68 ± 0.04 in the long lairage group; *p* < 0.05). It seemed that their excitability without having the opportunity to recover implied a significant depletion of muscle glycogen reserves with a profound effect on pH at 24 h post-mortem. Stressors appear to be additive [100], so that multiple stressors without the opportunity to recover during lairage resulted in a greater elevation of muscle pH. At the commercial level, the last pH measurement taken is one of the most important reference values to measure meat quality and is related to the depletion of glycogen reserves and the release of lactate caused by stressful handling [63]. It is also the most used instrumental indicator in studies that evaluate preslaughter handling, because it takes into account metabolic routes and muscle energy stores [11]. The Uruguayan National Beef Quality Audit estimated that pH higher than 5.8 costs the Uruguayan cattle industry 16.5 million of dollars annually, with 48% total losses of the meat chain [97].

The digestive process has a longer lag phase when animals are pasture-based fed [14,94]. In the present experiment, animals from the long lairage, ruminated during the night, thus, glycogen levels after 15 h could probably have been an important component of glucose availability. They probably had the opportunity to rest overnight when the environment of the slaughterhouse was quieter and could also have achieved some control over possible stress-induced energy intake caused by the new environment. In addition, these animals could have restored their muscle reserves from mobilized liver glucose and would have had greater time to restore muscle glycogen from gluconeogenesis during the resting period [14]. A similar experiment developed in Uruguay, with steers fed on pasture and comparing 3 vs. 12 h in lairage after 1.5 h of transport, did not find differences in pH values between lairage groups, but the glycogen content was lower in the short lairage group. This lower glycogen content was not enough to affect quality, but being consistent to the present study, it suggested a higher level of stress in the short lairage group, becoming a warning flag regarding animal welfare and higher risk regarding meat quality [14].

In addition of being used as “iceberg” or “key” welfare indicators during meat inspection, as a means of assessing and ensuring overall animal welfare from the farm of origin to the abattoir [11,101,102], is important to emphasize that bruises and the carcass pH values above 5.8, imply 85% of total economic losses in the Uruguayan meat chain [97]. Therefore, being an exporting country, it is mandatory for Uruguay both from an ethical and economic point of view to strengthen corrective actions and educational strategies throughout the entire chain, as well as to develop research initiatives to minimize the incidence of both problems. In this context, and from the present experiment results, the preslaughter lairage time of 15 h seems to be better than the shorter period of 3 h.

Temperament and pH.

In the present study, TIndex was not related to final pH values, not being consistent with Lensink et al. [72], who indicated that excitable animals may be most susceptible to stress generated by routine handling practices, such as loading and unloading, transport, and the new environment in the abattoir, reducing the muscle glycogen level in vivo [103] because of energy expenditure due to physical exercise or psychological stress, which may, in turn, increase the ultimate pH of muscles [104].

## 4. Conclusions

Considering the average daily gains, environmental conditions, animal health performance and mortality rate, it is possible to make the preliminary inference that animal welfare was not compromised in any diet during the finishing period. Due to the positive effect of temperament on productivity and on all physiological indicators at different preslaughter stages, it should be given paramount importance when talking about animal welfare. The psychological stress response of transportation may be minimized in 3.5-h travels by using best management practices, even with animals coming from extensive conditions. Increases in energy demands are unavoidable in fasting animals, especially with longer lairage, but adequate conditions and a calm environment may allow cattle to rest and recover while waiting in lairage pens until 15 h, with positive effects on the animal welfare and carcass quality. The emotional involvement or the psychological stress response did not differ between the contrasting lairage times evaluated, but the insufficient resting period from the short lairage in this experiment contributed to glycogen depletion and higher pH values. According to the experiment results, with pasture-based animals not fasting on the farm and after a short time of transportation (3.5 h), a longer preslaughter resting time (15 vs. 3 h) is desirable from the animal welfare perspective. Furthermore, the results suggest that this longer resting period would be also more convenient from the carcass quality perspective, with its consequent positive effects on the meat quality. Based on our results, international organizations should consider different realities and, therefore, contextualized scientific information when writing worldwide regulations or recommendations, as suggested by Costa [14].

## Figures and Tables

**Table 1:** CPK (log) values at different times, within each slaughter group. Least-square means ± Standard error.

Log CPK (U/L)	Time A Basal Value	Time B after Transportation	Time C after Lairage	Time D at Slaughter
3 h	1.99 ^c^ ± 0.50	2.48 ^b^ ± 0.51	2.39 ^b^ ± 0.52 b	2.71 ^a^ ± 0.51
15 h	2.08 ^c^ ± 0.50	2.44 ^b^ ± 0.51	2.40 ^b^ ± 0.51 b	2.67 ^a^ ± 0.50

Values with different letters in the same line differ *p* < 0.05.

**Table 2:** NEFA values at different times within each slaughter group. Least-square means ± Standard error.

NEFA (mmol/L)	Time A Basal Value	Time B after Transportation	Time C after Lairage	Time D at Slaughter
3 h	0.36 ^d^ ± 0.02	0.55 ^b^ ± 0.03	0.48 ^b,c,d^ ± 0.08	0.43 ^c,d^ ± 0.03
15 h	0.37 ^d^ ± 0.02	0.49 ^b,c^ ± 0.03	0.68 ^a^ ± 0.04	0.52 ^b^ ± 0.03

Values with different letters in the same line, differ *p* < 0.05.
